# Severe Immune Thrombocytopenic Purpura Associated With Acute Epstein–Barr Virus Infection: A Case Report

**DOI:** 10.7759/cureus.101517

**Published:** 2026-01-14

**Authors:** Inês Fiúza M. Rua, Sérgio Cabaço, Diogo Ramos, Wendy Moniz, Conceição Loureiro

**Affiliations:** 1 Internal Medicine, Unidade Local de Saúde São José, Lisbon, PRT

**Keywords:** ebv-associated immune thrombocytopenia purpura, epstein-barr virus, epstein-barr virus infections, immune thrombocytopenic purpura (itp), infectious mononucleosis, mucocutaneous bleeding, secondary thrombocytopenia, severe thrombocytopenia, thrombocytopenia

## Abstract

Immune thrombocytopenic purpura (ITP) is an autoimmune disorder characterized by immune-mediated platelet destruction. Viral infections, particularly Epstein-Barr virus (EBV), are recognized triggers of secondary ITP in children and young adults. Although EBV-associated ITP is often mild and self-limited, severe thrombocytopenia with clinically significant bleeding may occur.

We report the case of an 18-year-old previously healthy woman who presented with severe thrombocytopenia associated with mucocutaneous bleeding, lymphadenopathy, and serological evidence of acute EBV infection. The patient was diagnosed with EBV-induced ITP and was successfully treated with systemic corticosteroids, achieving complete hematological and clinical recovery. This case highlights the importance of considering EBV infection in young patients presenting with acute severe thrombocytopenia and bleeding manifestations, as well as the generally favorable prognosis with appropriate management.

## Introduction

Immune thrombocytopenic purpura (ITP) is an autoimmune disorder characterized by isolated thrombocytopenia resulting from immune-mediated peripheral platelet destruction. In immunocompetent children and young adults, ITP frequently develops following viral infections, including influenza virus and SARS-CoV-2; however, Epstein-Barr virus (EBV) is the most commonly implicated pathogen, particularly in adolescents and young adults [1].

Primary EBV infection typically manifests as infectious mononucleosis, with clinical features including fever, lymphadenopathy, pharyngitis, and, in some cases, hepatosplenomegaly [2]. Although the disease course is usually self-limited, hematological abnormalities may occur, such as lymphocytosis with atypical lymphocytes, mild thrombocytopenia, autoimmune hemolytic anemia, and, less frequently, ITP [1, 3-7]. The development of ITP is thought to result from EBV-induced immune dysregulation, including mechanisms such as molecular mimicry and polyclonal B-cell activation leading to the production of anti-platelet antibodies [6,8].

The presence of isolated thrombocytopenia in the absence of systemic disease, pancytopenia, or dysplastic features, together with serological evidence of acute EBV infection, supports the diagnosis of secondary ITP [8,9]. EBV-associated ITP generally follows a benign course with mild thrombocytopenia, often manifesting as petechiae or purpura; however, severe cases with clinically significant hemorrhagic complications have been reported [1,2,4,5].

Most patients experience favorable outcomes with supportive care or immunosuppressive therapy. Corticosteroids and intravenous immunoglobulin (IVIG) remain the main therapeutic options in symptomatic patients or those with significant bleeding [2-5,10]. In selected cases, intravenous anti-D immunoglobulin is also recommended as a first-line treatment option in RhD-positive, non-splenectomized patients, both children and adults [11].

## Case presentation

An 18-year-old woman with no relevant medical or surgical history, no regular medication, no known allergies, or significant epidemiological exposure presented to the emergency department with a four-day history of asthenia and odynophagia, treated with ibuprofen and amoxicillin without clinical improvement. She additionally reported epistaxis, generalized ecchymoses, and heavy menorrhagia lasting 10 days. Progressive cervical lymphadenopathy over the preceding three weeks was also noted.

On physical examination, the patient was subfebrile (37.7°C) and hemodynamically stable. Multiple ecchymoses and petechiae were observed on the upper and lower limbs. Oropharyngeal examination revealed hyperemic mucosa and hypertrophic tonsils with purulent exudate. Palpation identified multiple enlarged lymph nodes in the cervical, submandibular, occipital, retroauricular, supraclavicular, and axillary regions.

Initial laboratory evaluation revealed severe thrombocytopenia (6 × 10⁹/L) and anemia (hemoglobin 8.8 g/dL), with microcytic and hypochromic indices consistent with iron deficiency (serum iron 16 µg/dL, transferrin saturation 18.4%, ferritin 14.7 ng/mL). The total leukocyte count was normal (7.16 × 10⁹/L), with marked lymphocytosis (5.31 × 10⁹/L; 74.2%) and neutropenia (1.08 × 10⁹/L; 15.0%). Peripheral blood smear examination showed lymphocytosis in the leukocyte series, with lymphocytic pleomorphism and the presence of reactive (stimulated) lymphocytes; the platelet series showed thrombocytopenia, without platelet clumping; the erythrocyte series showed no significant morphological abnormalities. Liver biochemistry showed cytolysis, with elevated aminotransferases (aspartate aminotransferase up to 134 U/L and alanine aminotransferase up to 292 U/L), and mildly increased lactate dehydrogenase (365 U/L), and normal bilirubin and haptoglobin levels. C-reactive protein was negative.

Infectious serological testing revealed positive heterophile antibodies. EBV serology demonstrated positivity for viral capsid antigen IgM and IgG, positivity for early antigen IgG, and negativity for EBV nuclear antigen IgG, consistent with acute EBV infection. Serological tests for hepatitis B virus, hepatitis C virus, HIV-1/2, and cytomegalovirus were negative.

Given the severity of thrombocytopenia and the presence of neutropenia, key differential diagnoses, including drug-induced thrombocytopenia related to recent ibuprofen and amoxicillin exposure, hemophagocytic lymphohistiocytosis, and primary bone marrow disorders, were considered. However, the patient’s classic clinical presentation of infectious mononucleosis, the absence of pancytopenia or dysplastic features on peripheral blood smear, and the marked lymphocytosis with reactive morphology made these diagnoses less likely, guiding a decision to defer bone marrow examination initially.

**Figure 1:**
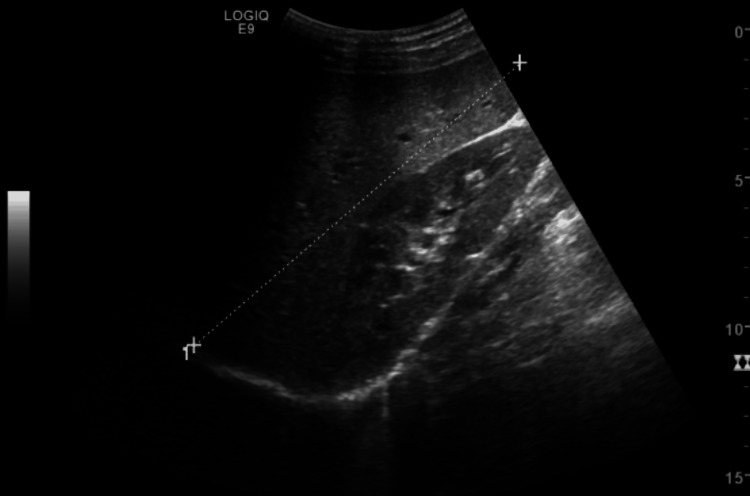
Abdominal ultrasound revealed moderate splenomegaly (154.8mm), with homogeneous parenchyma.

Abdominal ultrasound revealed moderate splenomegaly (154.8 mm) with homogeneous parenchyma (Figure 1), confirmed by abdominal computed tomography.

**Figure 2:**
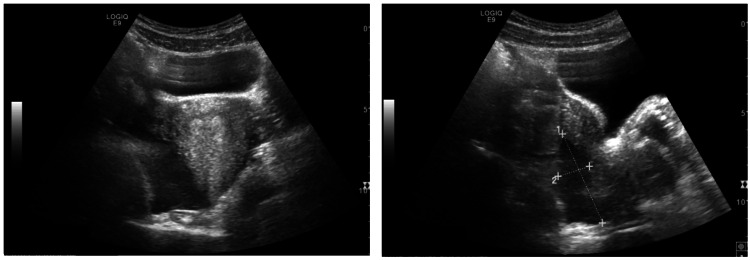
Pelvic ultrasound showed a normal-sized uterus with endometrial thickening (22 mm), normal ovaries, and a moderate amount of complex free fluid in the pouch of Douglas, measuring 55 × 18 mm in the mid-sagittal plane.

Given the presence of significant menorrhagia in the context of severe thrombocytopenia and anemia, a pelvic ultrasound was performed to evaluate for potential gynecological sources of bleeding and to exclude structural pelvic pathology. Pelvic ultrasound showed a normal-sized uterus with thickened endometrium (22 mm), normal ovaries, and a moderate amount of non-purulent free fluid in the Douglas pouch (Figure 2). 

A diagnosis of EBV-induced ITP was established, and treatment with oral prednisolone at a dose of 1 mg/kg/day was initiated. The associated iron-deficiency anemia was attributed to prolonged menorrhagia, with no laboratory evidence of hemolysis or bone marrow failure. Accordingly, a single dose of 1,000 mg of intravenous iron was administered. During hospitalization, the patient exhibited progressive clinical and laboratory improvement, with recovery of platelet counts and resolution of hemorrhagic manifestations.

During outpatient follow-up, complete normalization of blood counts and liver enzymes was observed (Table 1).

**Table 1:** Laboratory data during follow-up AST: aspartate aminotransferase; ALT: alanine aminotransferase; ALP: alkaline phosphatase; GGT: gamma-glutamyl transferase; LDH: lactate dehydrogenase

Parameter (units)	Admission (ED)	Day 2	Day 4	Day 9	Two months (Outpatient)	Reference range values
Hemoglobin (g/dL)	8.8	9.2	9.1	11.1	12.4	12.0-15.0
Leukocytes (×10⁹/L)	7.16	7.51	5.87	9.91	6.51	4.5-11.0
Lymphocytes (×10⁹/L)	5.31	3.64	3.46	3.43	2.45	0.9-3.5
Neutrophils (×10⁹/L)	1.08	1.99	1.73	5.17	3.12	2-8.5
Platelets (×10⁹/L)	6	92	111	128	170	150-450
AST (U/L)	65	134	43	13	16	5-34
ALT (U/L)	100	292	86	32	21	0.0-55
ALP (U/L)	57	82	75	62	54	40-150
GGT (U/L)	40	63	40	27	18	9-36
LDH (U/L)	365	302	216	152	106	125-220

Follow-up imaging demonstrated regression of hepatosplenic involvement and normalization of pelvic ultrasound findings.

## Discussion

ITP is a well-recognized hematological complication of acute EBV infection, particularly in children and young adults [1-3]. Its pathophysiology is thought to involve EBV-induced immune dysregulation, leading to immune activation, autoantibody production against platelet antigens, and increased splenic platelet clearance. Specific proposed mechanisms include molecular mimicry, where epitopes on EBV glycoproteins (e.g., gp350) cross-react with platelet surface glycoproteins such as GPIIb/IIIa. In addition, EBV drives polyclonal B-cell activation and immune complex formation, facilitating Fc receptor-mediated phagocytosis of antibody-coated platelets, predominantly within the spleen. The observed splenomegaly in this case is not only a sign of infection but also central to this pathogenic process, acting as a major site for both EBV reservoir and platelet clearance [2,6,12].

The marked lymphocytosis with reactive morphology observed in this patient reflects EBV-driven T-cell activation, while the transient elevation of aminotransferases is consistent with hepatic involvement due to infiltration by activated lymphocytes, a well-recognized feature of acute EBV infection [6,7].

Although EBV-associated thrombocytopenia is frequently mild, with a reported prevalence ranging from 25% to 50%, severe thrombocytopenia with platelet counts below 50 × 10⁹/L and clinically significant bleeding is rare, occurring in approximately 1.5% of hospitalized patients with EBV infection, according to published series [4,5,13,14]. The severe presentation observed in our patient, therefore, represents an uncommon but clinically important manifestation.

Diagnosis relies on the exclusion of alternative causes of thrombocytopenia and confirmation of acute EBV infection through compatible clinical features and serological testing [1-4,7]. In this case, the absence of pancytopenia, preserved red blood cell morphology, normal bilirubin and haptoglobin levels, and the rapid hematological response supported a diagnosis of peripheral platelet destruction rather than bone marrow failure or hemophagocytic lymphohistiocytosis [8,9]. Bone marrow examination is therefore typically unnecessary in young patients with a compatible clinical picture and prompt response to therapy [1,2].

Although iron deficiency secondary to prolonged menorrhagia was considered the primary cause of anemia in this patient, mild elevation of lactate dehydrogenase may reflect nonspecific cellular turnover during acute EBV infection; however, normal bilirubin and haptoglobin levels argued against clinically significant hemolysis [7,12].

Therapeutic management depends on the severity of thrombocytopenia and the presence of active bleeding. Corticosteroids and IVIG are recommended in cases of severe thrombocytopenia or hemorrhagic manifestations [1,4]. Although intravenous anti-D immunoglobulin is currently recommended as a first-line treatment option for ITP in RhD-positive, non-splenectomized patients, both children and adults, its use requires careful consideration. In the setting of acute EBV infection, anti-D Ig has been associated with an increased risk of severe hemolysis and acute renal failure [10,11]. Furthermore, the presence of concurrent hepatic involvement in this case supported the choice of corticosteroid therapy over IVIG, given reports of IVIG-associated drug-induced liver injury [8,15]. Given these safety concerns, systemic corticosteroid therapy was favored in the present case, resulting in a rapid and sustained hematological response.

The patient described in this report demonstrated progressive normalization of platelet counts and complete clinical recovery. This favorable outcome is consistent with the generally good prognosis reported in immunocompetent young patients with EBV-associated ITP. Nevertheless, the clinical course may be heterogeneous, and cases of persistent or relapsing thrombocytopenia have been described, underscoring the importance of appropriate follow-up [1-5].

## Conclusions

EBV-induced ITP should be considered in immunocompetent young patients presenting with acute severe thrombocytopenia, mucocutaneous bleeding, lymphadenopathy, and clinical features suggestive of infectious mononucleosis. Although the disease course is typically benign, severe thrombocytopenia with significant hemorrhagic risk may occur. Early recognition, exclusion of alternative etiologies, and timely initiation of appropriate therapy are essential to ensure a favorable prognosis. This case illustrates that severe, life-threatening thrombocytopenia can be the presenting feature of acute EBV infection. A comprehensive diagnostic approach must rigorously exclude other causes of cytopenias, including HLH and drug-induced effects, even in a classic clinical setting. Understanding the specific immune mechanisms at play informs therapeutic choices and highlights the importance of monitoring for both acute resolution and potential long-term sequelae.
